# Malnutrition affects infection rates and quality of life in patients undergoing primary hip and knee arthroplasty: a retrospective study

**DOI:** 10.5194/jbji-11-139-2026

**Published:** 2026-03-04

**Authors:** Domenico De Mauro, Chiara Comisi, Valeria Maccauro, Giovanni Balato, Sebastian Meller, Martina Di Martino, Fabiana Arduini, Giulio Maccauro, Raffaele Vitiello

**Affiliations:** 1 Department of Orthopedics and Geriatrics Sciences, Università Cattolica del Sacro Cuore, 00168, Rome, Italy; 2 Department of Orthopedics, Ageing and Rheumatological Sciences, Fondazione Policlinico Universitario A. Gemelli IRCCS, 00168, Rome, Italy; 3 Department of Public Health, Federico II University, 80131, Naples, Italy; 4 Department of Medical and Surgical Sciences, Nutritional Disorders unit, Fondazione Policlinico Universitario A. Gemelli IRCCS, 00168, Rome, Italy; 5 Department of Translational Medicine and Surgery, Università Cattolica del Sacro Cuore, 00168, Rome, Italy; 6 Charité-Universitätsmedizin Berlin, Corporate Member of Freie Universität Berlin, Humboldt-Universität zu Berlin and Berlin Institute of Health, Center for Musculoskeletal Surgery (CMSC), Berlin, Germany; 7 Infectious Diseases Unit, Department of Health Management, AORN Antonio Cardarelli, Naples, Italy; 8 Department of Chemical Science and Technologies, University of Rome “Tor Vergata”, via della Ricerca Scientifica, 00133, Rome, Italy

## Abstract

**Background**: Although malnutrition is globally associated with incremental morbidity, mortality, and cost, there has been a fundamental lack of consensus on diagnostic criteria for application in clinical settings. The study aims were (i) to identify the role of malnutrition as an independent risk factor for complications in the total joint arthroplasty (TJA) context; and (ii) to analyse the impact of malnutrition on treatment success, specifically in terms of patient-reported quality of life. **Methods**: The study retrospectively analysed 1070 patients undergoing total hip or knee arthroplasty between January 2019 and July 2022. Before the surgery, patients' nutritional status were assessed through Nutritional Risk Screening 2002 (NRS-2002) and controlling nutritional status (CONUT) scores. Patients were classified in two different groups: patients with NRS ≥ 3 were included in the Malnutrition group, and patients with NRS < 3 in the Control group. **Results**: A total number of 682 patients was included in the study. Mean age was 70.1 ± 9.6, 53 % women ratio, BMI 28.1 ± 4.8 kg m^−2^. A univariate regression, adjusted for age and sex, was performed. Both NRS-2002 and the CONUT score were significant risk factors in infectious complications and in EQ5 domains of quality-of-life assessment ( p<0.05 ). **Conclusions**: Malnutrition, as identified by both the NRS-2002 and CONUT scores, emerges as an independent risk factor, contributing to worse post-operative outcomes and increased complications, thereby decreasing the potential benefits of TJAs. Pre-operative nutritional assessment and targeted intervention to address malnutrition can play a crucial role in improving clinical outcomes, quality of life, and complication rates.

## 1 Introduction

Post-operative outcomes in patients undergoing primary hip and knee arthroplasty are influenced by a multitude of factors, including surgical technique, patient comorbidities, and peri-operative assessment care (Nwachukwu et al., 2015). Among these, nutritional status holds a critical role in optimizing surgical outcomes and preventing post-operative complications, especially infectious ones (Festa et al., 2024b). Malnutrition, defined as a state of nutrient deficiency or imbalance due to many different causes (Cederholm et al., 2019), is a common condition affecting approximately 30 %–50 % of the patient population (Khalatbari-Soltani and Marques-Vidal, 2015). Patients with poor nutritional status are more likely to suffer from adverse outcomes, have an elevated risk of mortality and morbidity, and experience significant socioeconomic implications (Festa et al., 2024a; Hiesmayr et al., 2009). Moreover, malnutrition is considered a significant, modifiable risk factor that is associated with an increase in morbidity and mortality rates, extension of length of stay (LOS), delayed wound healing, and higher rates of surgical complications (Azzolino and Lucchi, 2023; Fu et al., 2016). In fact, inadequate nutrient intake impairs immune function, reduces protein synthesis, and compromises muscle mass and tissue repair, leading to a weakened ability to recover from the physiological stress of surgery, a greater likelihood of reoperation, increased healthcare costs, and higher mortality rates (Hill et al., 1977).

Although malnutrition is globally associated with incremental morbidity, mortality, and cost, there has been a fundamental lack of consensus on diagnostic criteria for application in clinical settings (Cederholm et al., 2019). Emerging evidence underscores the importance of early nutritional assessment and intervention in the peri-operative period to mitigate these risks. A screening tool should be based on easy, reliable, and cost-effective measures and evaluations, in order to accurately identify patients with higher pre-operative risk factors. Additionally, thorough medical assessments prior to surgery, when followed by appropriate clinical interventions, have been demonstrated to significantly improve clinical outcomes (Golladay et al., 2016). Therefore, the involvement of a multidisciplinary team to conduct a comprehensive evaluation of the patients is essential for optimizing results and addressing their complex needs effectively.

Current literature presents several diagnostic criteria or indicators, including serologic laboratory values and standardized nutritional screening scores (Gu et al., 2019). The controlling nutritional status (CONUT) score is a novel, simple evaluation measure that is calculated using serum albumin level, total cholesterol concentration, and total lymphocyte count measurement (Ignacio de Ulíbarri et al., 2005). The Nutritional Risk Screening (NRS-2002) evaluates both the patient's nutritional status – based on weight loss, body mass index (BMI), and overall condition or food intake – and the severity of disease, which reflects stress metabolism (Hersberger et al., 2020). Both of these tools have been associated with a higher risk of adverse clinical outcomes when malnutrition is present.

Despite the availability of various diagnostic tools and indicators, no single approach has been universally accepted. The absence of a standardized definition complicates efforts to identify malnutrition consistently across different patient populations (Bruun et al., 1999) Establishing a universally recognized criterion would be crucial, in order to reduce the incidence of post-operative complications and mortality, particularly in vulnerable populations such as surgical in-patients.

This study aims were (i) to identify the role of malnutrition as an independent risk factor for complications in the total joint arthroplasty context; and (ii) to analyse the malnutrition impact on treatment success, specifically in terms of patient-reported quality of life.

## 2 Materials and methods

### 2.1 Study design and data collection

The study retrospectively analysed 1070 patients undergoing total hip or knee arthroplasty between January 2019 and July 2022 at third-level referral institutions. The observational study followed the STROBE guidelines (von Elm et al., 2007). The study adhered to the Declaration of Helsinki principles. No ethical approval was required according to the Local Ethics Committees, due to the retrospective and observational nature of the study.

Inclusion criteria were (i) patients diagnosed with primary osteoarthritis (OA); (ii) undergoing THA or TKA; and (iii) minimum 12 months follow-up.

Exclusion criteria were (i) THA or TKA implanted for secondary OA, femoral neck fracture or oncological lesions; (ii) lacking nutritional assessment; with (iii) incomplete data.

PJI diagnosis was made according to the 2018 Philadelphia International Consensus Meeting (ICM) criteria (Balato et al., 2019). The following data were recorded: demographic data; anthropometric measurements, such as BMI and the Charlson comorbidity index (CCI); clinical history, and surgical history.

According to the Nutritional Risk Screening (NRS) score, patients were classified in two different groups: patients with NRS ≥ 3 were included in the Malnutrition group, and patients with NRS < 3 in the Control group.

**Figure 1:**
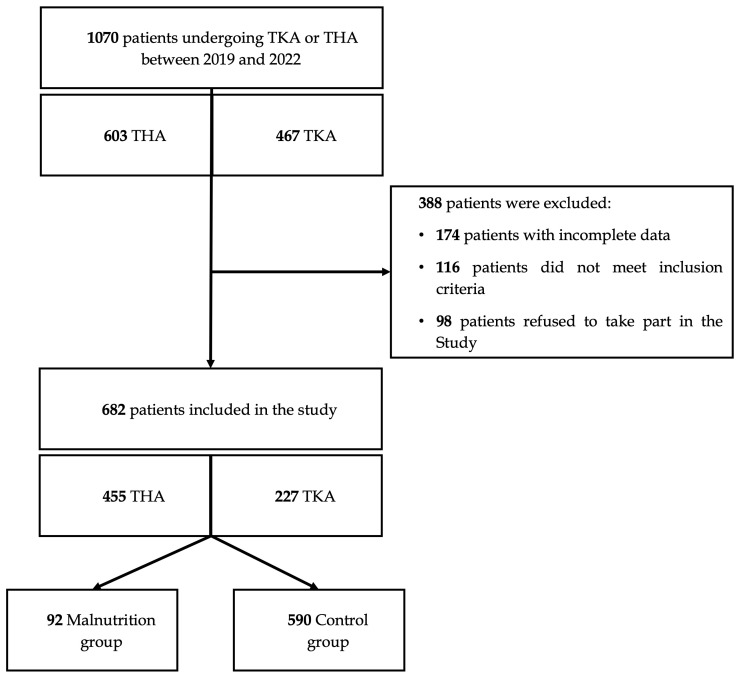
Flowchart for patient selection.

### 2.2 Assessment measures

Before the surgery, patients' nutritional status were assessed through the Nutritional Risk Screening 2002 (NRS-2002) (Kondrup et al., 2003) and Controlling Nutritional Status (CONUT) scores (Ignacio de Ulíbarri et al., 2005). NRS-2002 is calculated by assessing nutritional status, disease severity, and patient age. Nutritional status is determined by evaluating weight loss over the last 3 months (0 points for less than 5 % loss, 1 point for 5 %–10 % loss, and 2 points for more than 10 % loss), a reduction in food intake compared to usual requirements (0 points for less than 25 % reduction, 1 point for 25 %–50 % reduction, and 2 points for more than 50 % reduction), and BMI (0 points for a BMI ≥ 20.5, 1 point for a BMI between 18.5 and 20.5, and 2 points for a BMI less than 18.5). Disease severity is evaluated by assigning 1 point for mild clinical conditions and 2 points for moderate. A total score of 3 or more indicates a nutritional risk. The CONUT score, instead, assesses nutritional status using serum albumin, total cholesterol, and total lymphocyte count. Points are assigned based on thresholds: serum albumin ( > 3.5 g dL^−1^ = 0, 3.0–3.49 = 2, 2.5–2.99 = 4, < 2.5 = 6), total cholesterol ( > 180 mg dL^−1^ = 0, 140–180 = 1, 100–139 = 2, < 100 = 3), and lymphocyte count ( > 1600 mm^−3^ = 0, 1200–1599 = 1, 800–1199 = 2, < 800 = 3). The total score ranges from 0 to 12, with higher scores indicating worse nutritional status. A last follow-up was additionally provided a quality-of-life assessment through EuroQol 5 Dimensions (EQ5-5D) (Giesinger et al., 2015). It consists of five different subscales, assessing pain, mobility, daily activities, self-care, and mental health. A higher value corresponds to a worse outcome.

### 2.3 Data analysis

The data are reported as the mean and standard deviation for continuous variables and frequency distributions in percentages for categorical variables. Continuous variables underwent comparison through either the t student test or the Mann-Whitney U test, depending on the distribution of the data. Categorical variables were represented as proportions and subjected to comparison utilizing either the Fisher exact test or the chi-squared test. Logistic univariate regression, adjusted for age and sex, was performed to assess the role of malnutrition as a risk factor in terms of outcomes and quality of life. The significance was established for a p value < 0.05. Data analysis was donee with the SPSS 29.0 software program (SPSS, Inc., Chicago, IL, USA).

**Table 1:** Demographic and clinical data of the included patients.

	Malnutrition group ( n = 92)	Control group ( n = 590)	p value
0 Age (years) (med, IQR)	78.0 ± 9.0	70.0 ± 13.0	< 0.001^*^
Women ratio ( n , %)	50 (54.3 %)	312 (52,9 %)	0.793
BMI (kg m^−2^) (med, IQR)	26.6 ± 5.0	27.5 ± 5.9	0.016^*^
CCI (med, IQR)	5.0 ± 3.0	2.0 ± 3.0	< 0.001^*^
NRS-2002 (med, IQR)	3.0 ± 0.0	1.0 ± 1.0	< 0.001^*^
CONUT score (med, IQR)	3.0 ± 3.0	1.0 ± 2.0	< 0.001^*^
THA ( n , %)	67 (72.8 %)	388 (64.4 %)	0.181
TKA ( n , %)	25 (27.2 %)	202 (35.6 %)	0.181
Hospitalization (days) (med, IQR)	6.0 ± 4.0	5.0 ± 3.0	0.275
Follow-up (months) (med, IQR)	25.0 ± 24.0	26.0 ± 27.0	0.162
Outcomes			
Complications ( n , %)	4 (4.3 %)	14 (2.4 %)	0.271
Infections ( n , %)	3 (3.3 %)	3 (0.5 %)	0.009^*^
Dislocation ( n , %)	1 (1.1 %)	9 (1.5 %)	0.745
Re-intervention ( n , %)	4 (4.3 %)	14 (2.4 %)	0.271
Deaths ( n , %)	2 (2.2 %)	8 (1.4 %)	0.542
Quality of life (EQ5)			
Pain (med, IQR)	1 ± 0	1 ± 0	0.603
Mobility (med, IQR)	1 ± 1	1 ± 1	0.012^*^
Daily activities (med, IQR)	1 ± 1	1 ± 0	0.003^*^
Self-care (med, IQR)	1 ± 1	1 ± 0	< 0.001^*^
Mental health (med, IQR)	1 ± 0	1 ± 0	0.004^*^

^*^ significant p value < 0.05; n : number; SD: standard deviation; NSR: Nutritional Risk Screening; BMI: body mass index.

## 3 Results

A total number of 682 patients were included in the study. The flowchart of the patient selection process is shown in Fig. 1. Mean age was 70.1 ± 9.6, 53 % women ratio, BMI 28.1 ± 4.8 kg m^−2^. Demographic and clinical data of both groups are shown in Table 1.

**Figure 2:**
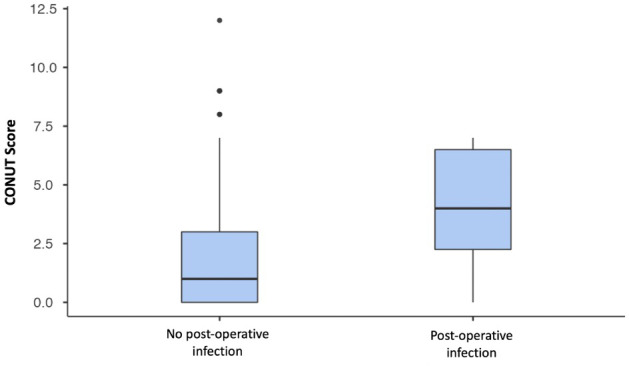
Boxplot comparing the CONUT (controlling nutritional status) scores between patients who developed post-operative infections and those who did not. The group with post-operative infections shows higher median CONUT scores, suggesting poorer pre-operative nutritional status compared to the non-infected group.

### 3.1 Malnutrition and complications

In terms of outcomes, there were no statistically significant differences in overall complication rates between the two groups ( p=0.271 ). However, the Malnutrition group demonstrated a higher rate of infectious complications at 3.3 %, compared to 0.5 % in the Control group ( p=0.009 ) (Fig. 2). Other complications, including the total number of dislocations and re-interventions, as well as mortality rates, did not significantly differ between the groups ( p > 0.05) (Table 1).

### 3.2 Malnutrition and quality of life

Quality of life in patients in the Malnutrition group was slightly but significantly worse than the Control group in all the subscales, except the pain subscale (Table 1).

As further analysis, a univariate regression, adjusted for age and sex, was performed, in order to deeper assess the role played by malnutrition in clinical outcomes and quality of life, analysing both NRS and CONUT scores as risk factors. The results are shown in Tables 2 and 3.

**Table 2:** Univariate regression, adjusted for age and sex, demonstrated a significant impact of the NRS-2002 on joint arthroplasty functional outcomes and quality of life, favouring the Control group. This finding underscores the role of malnutrition in affecting the infection rates, revision rates, and quality of life.

NRS-2002	Complications				EQ5				
	Infection	Dislocation	Revision	Death	Mobility	Pain	Activities	Self-care	Depression
p value	0.002	0.453	0.032	0.468	0.006	0.096	< 0.001	< 0.001	0.002
OR	3.31	1.27	1.63	1.27	2.74^*^	1.67^*^	3.68^*^	3.89^*^	3.10^*^
CI 95 %	1.56	0.68	1.04	0.67	0.03	- 0.01	0.06	0.06	0.02
	7.06	2.38	2.54	2.42	0.17	0.11	0.19	0.18	0.10

^*^ this value represents the t value, as linear regression was performed.

**Table 3:** Univariate regression, adjusted for age and sex, demonstrated a significant impact of the CONUT score on joint arthroplasty functional outcomes and quality of life, favouring the Control group. This finding underscores the role of malnutrition in affecting infection rates, mortality, and quality of life.

CONUT	Complications				EQ5				
	Infection	Dislocation	Revision	Death	Mobility	Pain	Activities	Self-care	Depression
p value	0.023	0.107	0.075	0.043	< 0.001	0.008	< 0.001	0.003	0.002
OR	1.39	1.25	1.20	1.27	3.96^*^	2.65^*^	3.66^*^	2.95^*^	3.18^*^
CI 95 %	1.05	0.95	0.98	1.01	0.03	0.01	0.03	0.01	0.01
	1.85	1.62	1.47	1.60	0.09	0.07	0.08	0.07	0.05

^*^ this value represents the t value, as linear regression was performed.

## 4 Discussion

The study shows how malnutrition indicators, both the NRS-2002 and CONUT scores, are significantly associated with quality of life and complications, particularly infectious ones in patients undergoing total joint arthroplasty. These findings highlight the critical impact of malnutrition on patient well-being and clinical outcomes after total knee and hip arthroplasty.

### 4.1 Malnutrition and complications

These factors can decrease the benefits of the surgery, delaying mobility improvements and pain relief that are expected from the procedure (Ihle et al., 2018; Roche et al., 2018). In addition, the significant findings from this study, recognizing malnutrition as a risk factor for increased infectious complications, can highlight the compromised immune function in malnourished individuals, increasing their susceptibility to infections (De Mauro et al., 2024b; Gu et al., 2019; Pes et al., 2023; Yi et al., 2015). Malnourished patients often have weakened and reduced muscle mass, and impaired wound healing, all of which can lead to longer recovery times, increased complications, and diminished functional outcomes post-surgery (Eminovic et al., 2021; Wilson et al., 2020). 

### 4.2 Malnutrition and quality of life

This study demonstrated that malnutrition, assessed using both the CONUT score and NRS-2002, also significantly impacts the quality of life (EQ5) of patients undergoing total joint arthroplasty, affecting all subscales of the quality-of-life measure, such as mobility, self-care, usual activities, pain/discomfort, and depression. This reduced quality of life directly influences the perceived benefits of TJA. This emphasizes the need for early detection and management of malnutrition to improve patient outcomes and reduce complication risks. Integrating comprehensive nutritional assessments into patient care is thus essential for addressing malnutrition and enhancing overall well-being (De Mauro et al., 2024a; Schroer et al., 2019). 

### 4.3 Malnutrition assessment

The literature shows a wide range of malnutrition prevalence among patients undergoing primary prosthesis surgery, from 3.7 % to about 56.0 %, influenced by differences in study populations and assessment methods (Gu et al., 2019). In contrast, our study, involving 682 patients, found a malnutrition prevalence of 15.6 %. This rate is lower than some higher estimates and shows a more moderate value. Most studies in the literature demonstrate significant correlations between serum marker values and morbidity, complications, hospitalization, and clinical outcomes, addressing these findings as malnutrition (Bohl et al., 2016; De Mauro et al., 2023, 2025; Fu et al., 2017; Roche et al., 2018; Rudasill et al., 2018; Walls et al., 2015). However, these serum markers alone do not provide direct and conclusive evidence of malnutrition. Alterations in markers such as albumin, prealbumin, and total leucocyte count, while commonly associated with adverse clinical outcomes, can be influenced by a range of factors including inflammation, infection, or other non-nutritional conditions (Jin et al., 2023). Eminovic et al. (2021) demonstrated that alterations in serum values, such as albumin levels and total leucocyte count, are observed in both malnourished and well-nourished patients. Our study's restrictive definition of malnutrition minimizes the inclusion of patients in malnutrition spectrum compared with non-specific serum values, reducing false positives (Blevins et al., 2018; Ellsworth and Kamath, 2016; Eminovic et al., 2021). This finding suggests that relying only on serum biomarkers may not provide a fully accurate assessment of nutritional status, as these changes can occur due to various factors unrelated to malnutrition. By combining serum-based assessments like the CONUT score with clinical and anthropometric evaluations from the NRS-2002, a more accurate identification of malnourished individuals was achieved. This rigorous methodology ensures that these findings offer a reliable reflection of malnutrition prevalence and its impact on quality of life and complications.

### 4.4 Strengths and limitations

Evaluating and addressing nutritional deficiencies before surgery can improve the surgical effectiveness, reduce the complication rate, and enhance recovery (Schroer et al., 2019). This approach not only benefits individual patients but also has positive implications for public health and the healthcare system by reducing overall healthcare costs (Bala et al., 2020; Rudasill et al., 2018). Therefore, integrating thorough nutritional assessments into pre-operative care is a valuable strategy for improving patient outcomes and promoting public health.

The strengths of this study include the robustness of the malnutrition assessment criteria, the homogeneity of the patient cohort, and the sample size (which is consistent with other similar studies on this topic in the literature). By using comprehensive and well-defined criteria for evaluating malnutrition, and by studying a relatively large and uniform patient population, our findings provide a reliable and relevant contribution to the field. However, the study also has limitations. First, its retrospective nature means that it relies on existing data, which can limit the depth of analysis and the ability to establish causality. In fact, potentially, several factors such as comorbidity burden, systemic inflammatory status, frailty, and socioeconomic status were not recorded in the current study, and they may act as confounder factors influencing the increased risk of infections in malnourished patients. Second, approximately 36 % of the initially selected cohort was excluded, primarily due to missing nutritional assessments or incomplete clinical data. Although this was necessary to ensure data completeness and reliability, this exclusion may introduce selection bias and limit the generalizability of the results. Third, while malnutrition is often considered a potentially modifiable risk factor, not all forms or causes of malnutrition are equally valuable to correction. The severity, etiology, and time course of malnutrition, as well as the feasibility and effectiveness of targeted nutritional interventions, may all influence post-operative outcomes. In addition, the absence of a longitudinal analysis of the impact of nutritional interventions over time means that we cannot fully assess how changes in nutritional status might affect long-term outcomes. These limitations suggest areas for future research, such as prospective studies with repeated assessments of nutritional status to better understand the dynamic relationship between nutrition and patient outcomes.

## 5 Conclusions

Malnutrition, as identified by both the NRS-2002 and CONUT scores, emerges as an independent risk factor, contributing to poor post-operative quality of life and increased infectious complications, thereby decreasing the potential benefits of TJAs. Although causality cannot be established due to the retrospective nature of the study, these findings demonstrate the importance of pre-operative nutritional assessment. Further prospective studies are necessary to determine whether targeted nutritional interventions can effectively modify this risk and improve clinical outcomes.

## Data Availability

The data that support the findings of this study are available from the corresponding author upon reasonable request. The data are not publicly available due to privacy and ethical restrictions.

## Appendix A Abbreviations

The following abbreviations are used in this paper:

**Table:**

BMI:	body mass index
CCI:	Charlson comorbidity index
CONUT:	controlling nutritional status
EQ5-5D:	EuroQol 5 Dimensions
ICM:	International Consensus Meeting
LOS:	length of stay
NRS:	Nutritional Risk Screening
PJI:	periprosthetic joint infection
STROBE:	STrengthening the Reporting of Observational Studies in Epidemiology
TJA:	total joint arthroplasty
THA:	total hip arthroplasty
TKA:	total knee arthroplasty
